# Novel treatment-specific causal biomarkers for colorectal cancer by omics integration

**DOI:** 10.1093/nargab/lqaf053

**Published:** 2025-06-19

**Authors:** Akram Yazdani, Azam Yazdani, Raul Mendez-Giraldez, Gianluigi Pillonetto, Esmat Samiei, Reza Hadi, Heinz-Josef Lenz, Alan P Venook, Ahmad Samiei, Andrew B Nixon, Joseph A Lucci, Scott Kopetz, Monica M Bertagnolli, Federico Innocenti

**Affiliations:** Division of Pharmacotherapy and Experimental Therapeutics, Eshelman School of Pharmacy, University of North Carolina at Chapel Hill, NC 27599, United States; McGovern Medical School, University of Texas Health Science Center at Houston, TX 7030, United States; Division of Preventive Medicine, Department of Medicine, Brigham & Women’s Hospital, Harvard Medical School, Boston, MA 02115, United States; Biostatistics and Computational Biology Branch, National Institute of Environmental Health Sciences, Durham, NC 27709, United States; Department of Information Engineering, University of Padova, Padova 35131, Italy; Gam Electronic, Tehran 45611, Iran; School of Mathematics, University of Science and Technology of Iran, Tehran 13114, Iran; USC Norris Comprehensive Cancer Center, Los Angeles, CA 90033, United States; University of California at San Francisco, San Francisco, CA 94158, United States; Division of Pulmonary Medicine, Boston Children's Hospital, Boston, MA 02115, United States; Duke Center for Cancer Immunotherapy, Duke University, Durham, NC 27701, United States; Department of Pharmacy, University of Texas Heath-Memorial Hermann Cancer Center, Houston, TX 77030, United States; Department of Gastrointestinal Medical Oncology, University of Texas M.D. Anderson Cancer Center, Houston, TX 77030, United States; Department of Surgical Oncology, University of Texas M.D. Anderson Cancer Center, Houston, TX 77030, United States; Dana-Farber/Partners Cancer Care, Harvard Medical School, Boston, MA 02215, United States; Division of Pharmacotherapy and Experimental Therapeutics, Eshelman School of Pharmacy, University of North Carolina at Chapel Hill, NC 27599, United States

## Abstract

While monoclonal antibody-based targeted therapies have substantially improved progression-free survival in cancer patients, the variability in individual responses poses a significant challenge in patient care. Therefore, identifying cancer subtypes and their associated biomarkers is required for assigning effective treatment. In this study, we integrated genotype and pre-treatment tissue RNA-seq data and identified biomarkers causally associated with the overall survival (OS) of colorectal cancer (CRC) patients treated with either cetuximab or bevacizumab. We performed enrichment analysis for specific consensus molecular subtypes (CMS) of CRC and evaluated differential expression of identified genes using paired tumor and normal tissue from an external cohort. In addition, we replicated the causal effect of these genes on OS using a validation cohort and assessed their association with The Cancer Genome Atlas Program data as an external cohort. One of the replicated findings was *WDR62*, whose overexpression shortened OS of patients treated with cetuximab. Enrichment of its overexpression in CMS1 and low expression in CMS4 suggests that patients with the CMS4 subtype may derive greater benefit from cetuximab. In summary, this study highlights the importance of integrating different omics data for identifying promising biomarkers specific to a treatment or a cancer subtype.

## Introduction

While targeted therapies utilizing monoclonal antibodies have improved progression-free survival in patients, the pursuit of more effective cancer treatments remains a top priority. What complicates this pursuit is the diverse range of patient responses to existing therapies, which manifests as a significant obstacle in understanding the underlying variability in clinical outcomes [1]. Therefore, the key to addressing this challenge is identifying cancer subtypes and their associated biomarkers, allowing for precision in treatment strategies and ultimately leading to enhanced patient outcomes and a personalized approach to cancer care.

In colorectal cancer (CRC), molecular classifications have provided valuable insights into disease biology. One such classification is the transcriptomics-based consensus molecular subtype (CMS), which categorizes CRC into four groups, CMS1 (microsatellite instability immune), CMS2 (canonical), CMS3 (metabolic), and CMS4 (mesenchymal), based on the underlying biology of the disease rather than relying solely on clinical outcomes [2, 3]. While these classifications have been instrumental in stratifying patients, the subtypes still exhibit molecular heterogeneity and do not fully capture the complexity of CRC. As a result, several key aspects of CRC progression and treatment response remain unresolved [4], highlighting the need to move beyond broad classifications to identify more precise molecular determinants that drive differential treatment responses in CRC. To address this gap, our study aims to identify pre-treatment biomarkers with a causal impact on overall survival (OS) in CRC patients, ultimately guiding precision treatment strategies. We hypothesize that gene expression profiles contribute to differential treatment responses. By integrating genomic and transcriptomic data, we seek to identify these biomarkers and elucidate their role in modulating treatment response.

To this end, we analyzed data from a randomized phase III trial (CALGB/SWOG 80405) involving 1284 patients with KRAS wild-type advanced or metastatic CRC. These patients were treated with either cetuximab or bevacizumab in combination with chemotherapy (FOLFOX or FOLFIRI). Cetuximab targets the epidermal growth factor receptor and influences immune cells, including natural killer cells and macrophages [5], while bevacizumab targets vascular endothelial growth factor (VEGF) and inhibits tumor angiogenesis. Although the clinical outcomes of this study did not reveal a significant difference in OS between these two treatments when used as first-line therapy [6], the distinct biological pathways they affect suggest that different molecular mechanisms underlie treatment response and resistance. Hence, investigating these mechanisms at the molecular level is crucial to identifying predictive biomarkers and improving precision treatment strategies.

We analyzed gene expression (RNA-seq) data to investigate their influence on treatment response, segmenting our dataset into a discovery cohort and a validation cohort based on gene expression availability (Supplementary Fig. S1 and Supplementary Table S1). In the discovery cohort, comprising 273 samples with RNA-seq data (Supplementary Fig. S2), we conducted one-sample Mendelian randomization (MR) analysis, integrating germline genotype, RNA-seq, and treatment-specific OS data. In the validation cohort, which included 602 patients with available germline genotype data and clinical outcomes, including OS, we performed a two-sample MR analysis to replicate the results obtained in the discovery cohort. To further validate our results, we incorporated external cohorts, including The Cancer Genome Atlas (TCGA) data and the GSE146889 dataset sourced from the Gene Expression Omnibus database. In addition, we examined the association between the identified biomarkers and CMSs to refine our understanding of their role in modulating treatment response across distinct patient groups. This integrative approach allowed us to link molecular features with clinical outcomes, providing insights into the biological mechanisms driving differential responses to therapy and aiding in the development of precision oncology strategies.

## Materials and methods

### Ethical statement

While institutional review board approval was required at all participating centers and all participating patients provided written informed consent [6], this study has used deidentified samples.

### Data

Patients in this study were drawn from the Cancer and Leukemia Group B (CALGB; now a part of the Alliance for Clinical Trials in Oncology) and SWOG 80405 (Alliance) trial. The trial was initiated in September 2005 with a total of 2326 patients randomized to the three treatment arms (bevacizumab, cetuximab, or their combination in addition to chemotherapy with FOLFIRI or FOLFOX).

#### Genotyping

DNA was extracted from peripheral blood. The first genotyping batch was performed on the Illumina HumanOmniExpress-12v1 platform at the Riken Institute (Tokyo, Japan) and included 731 412 genotyped variants. The second genotyping batch was performed on the Illumina HumanOmniExpress-8v1 and included 964 193 single nucleotide polymorphisms (SNPs). A total of 719 461 SNPs from HapMap from batch 1 were also on the chip from batch 2. The quality control was performed to remove SNPs with mismatched annotation between the two platforms, genotyping call rates <99%, departure from Hardy–Weinberg equilibrium (*P-value* < 10^−8^), allele frequencies <0.05, and individuals with genotyping call rate <0.90. Passing the filters, 540 021 SNPs genotyped for 1165 samples remained [7].

#### Tumor RNA sequencing

Tumor RNA was extracted from formalin-fixed paraffin-embedded tumor blocks (96% primary, 2% metastatic, and 2% unknown) from 584 CALGB/SWOG 80405 patients at the baseline. TruSeq RNA Access target enrichment and library preparation protocol was performed using 250 ng of template RNA. Sequencing was done using synthesis chemistry targeting 50 million reads with a read length of 2 × 100 bp per sample on the HiSeq 2500. Data processing was conducted using standard procedures [8].

#### Clinical outcomes and covariates

The primary endpoint of OS was calculated from the time of study entry to death or last known follow-up for those without reported death. The median follow-up duration for the 640 samples in the bevacizumab and cetuximab arms was 65.7 months (95% confidence interval, 63.5–70). In addition, BRAF V600E and all RAS mutation status were determined by BEAMing (beads, emulsion, amplification, magnetics; Hamburg, Germany) technology [9] and included in the analysis as covariates in addition to age and gender.

### Data preprocessing

Among 584 samples with RNA-seq data, 86% were European American, 9% African American, and 5% from other ethnicities. Therefore, we focused on primary tumor samples from European American to avoid analysis being confounded due to population stratification. We excluded genes with low expression variation across samples (standard deviation <0.5) and genes with low counts across the samples (>30% zeros). The remaining 8301 genes were used for the analysis, and upper quartile normalization was applied to make gene expression values comparable across different samples. We removed duplicated samples (*n* = 5) and tumors with low gene expression across the genome (>50% genes with zero counts, *n* = 1). We then transformed the RNA-seq data into the log_2_ scale for the analysis. We performed principal component analysis to assess for any potential technical and systematic variation or hidden population stratification in RNA-seq data (Supplementary Fig. S3). To verify the self-reported gender, we applied *k*-mean clustering using the expression of genes in chromosome Y, resulting in five samples with mismatched biological gender and recorded gender [10] (Supplementary Fig. S4).

We used 1055 tumor samples (European American), genotyped at 540 021 SNPs for imputation. We used phased haplotypes from the Haplotype Reference Consortium (HRC) panel through the Michigan server [11]. Phasing was done using the Eagle v2.4 algorithm [12]. The HRC panel combines sequence data across >32 000 individuals from >20 medical sequencing studies. Through imputation, we generated genotypes for additional SNPs, and the final set of 5 539 144 common SNPs was selected based on imputation score (>0.7) and minor allele frequency (MAF) (>0.05). These imputed SNPs were used in all subsequent analyses.

### Immune cell type abundance

Since the RNA-seq data in this study have been generated from heterogeneous tumor samples composed of multiple cell types, correcting for the abundance of different cell types in the data is crucial to avoid the analysis being confounded. We estimated the abundance of immune cell types in our RNA-seq data using CIBERSORTx [13] with the validated leukocyte gene signature matrix as a reference. We defined a cell phenotype to be enriched in our data if at most 30% of its estimated scores across samples are zero and its standard deviation is >0.12. As a result, nine hematopoietic cell phenotypes were enriched in our data: naive and memory B cells, plasma cells, CD8^+^ T cells, resting and activated memory CD4^+^ T cells, M0 and M2 macrophages, and activated mast cells [10]. These enriched cell type abundances data (Supplementary Table S1) were used to adjust the gene expression levels in our downstream analysis.

### *cis*-eQTL analysis

To identify germline genetic variants associated with tumor gene expression, we previously focused on *cis*-eQTL [14]. For all pairs of genes and SNPs within 1 Mb upstream and downstream of the gene’s transcription start site, we applied a linear regression model while accounting for covariates (gender, age, BRAF V600E mutations and all RAS mutation status, batch effects, and enriched cell type abundances). We performed *cis*-eQTL mapping using FastQT [15]. We applied the adaptive permutation mode of FastQTL while setting it for 10 000 permutations. Out of 8301 genes analyzed, 352 were identified with at least one *cis*-eQTL at the gene level, meeting the threshold of an adjusted *P*-value <.05. This analysis, which evaluated the relationship of 33 209 829 *cis*-eQTL-gene pairs, has been published elsewhere [16] (Supplementary Figs S5 and S6). Hereafter, for simplicity, we use eQTL to refer to *cis*-eQTL.

### MR study

We conduct one-sample MR analysis to identify gene expressions that have a causal relationship with OS, using genetic factors as instrumental variables (IVs). In this analysis, we focused on genes with eQTLs and considered these eQTLs as potential IV candidates. We then take the following steps to ensure that the assumptions required for IVs are satisfied.

- *Gene–OS association*: To investigate whether genes (*G*) with eQTL are associated with OS (i.e. the outcome), we applied a multivariable time-variant additive hazard regression model.
  \begin{eqnarray*}
h\left( {t{\mathrm{|}}X} \right) = {h_0}\left( t \right) + \gamma {\left( t \right)^{T}}Z\ + \theta {\left( t \right)^{T}}E + \beta {\left( t \right)^{T}}G.
\end{eqnarray*}

Here, $h( {t{\mathrm{|}}X} )$ is the hazard function, ${h_0}( t )$ is the baseline hazard, and $\beta ( t )$ is a $p \times 1$ time-varying coefficient vector. The model accounts for the set of covariates (*Z*) including gender, age, BRAF V600E mutations, and all RAS mutation status and $\gamma ( t )$ is a time-varying coefficient vectors for covariates. In addition, since our RNA-seq data is bulk, the presence of various immune cell types in the tumor microenvironment may influence gene expression profiles, potentially introducing bias. To address this, the model adjusts for the abundances of immune cell types (*E*) to minimize this bias while $\theta ( t )$ represents their time-varying coefficient. This adjustment ensures that the associations between gene expression and OS more accurately reflect the tumor cells themselves, rather than being confounded by immune cell contributions.

This additive hazard regression model relaxes the assumption of the time-invariant effect of the genes on OS and allows for estimating causal effects [17]. Using this model, we conducted separate analysis for the patients under bevacizumab treatment and cetuximab treatment. To enhance the feasibility of conducting multivariable analysis and to prevent overfitting, we employed *k*-means clustering and clustered all genes in four clusters [16]. Using these clusters, we divided the 352 genes into four groups and conducted multivariable analyses for each group, incorporating all genes with eQTLs within each cluster into the corresponding model. Genes associated with OS (*P*-value <.1) were considered for causal study using the MR technique reviewed in the next steps.

- *Assessment of pleiotropy absence*: We conducted a genome-wide association study (GWAS) and estimated the effect of IVs on OS via a Cox proportional hazard model while adjusting for covariates gender, age, tumor location, all-RAS, and KRAS mutation, as well as the first principal component accounting for the batch effect (Supplementary Fig. S7). The significance threshold in GWAS is typically set at a *P*-value of <5 × 10^−8^. Any association above this threshold for IV-OS associations can be assumed as a lack of pleiotropy. However, we employed a stringent threshold of *P*-value <1 × 10^−4^ and excluded IVs with *P*-values <1 × 10^−4^ to ensure that the assumption of lack of pleiotropy holds.
- *Gene–OS causal association:* We evaluated the causal impact of associated genes with OS by predicting the expression level of each gene using its IVs. If the predicted expression value is associated with OS, the corresponding gene is considered as a putative cause of OS.

To predict the expression level of a gene, we selected SNPs associated with 352 genes from eQTL analysis, using a nominal *P*-value threshold of <.05. The number of eQTLs can vary from just a few to several dozen across different genes. To generate independent IVs, we initially clustered the SNPs of the gene based on their pairwise correlation (*r*^*2*^ > 0.1 within each cluster) using hierarchical clustering. We then selected one IV from each cluster as a representative proxy to capture the genetic variation within the cluster. By selecting one IV as the proxy, we ensured that the chosen IVs for a gene were independent while reducing redundancy and minimizing the risk of overfitting in the predictive model. We then predicted the expression level of each gene ($\hat{g}$) as follows:


\begin{eqnarray*}
{\hat{g}_i} = \ {Q_i}\ {W_i}
\end{eqnarray*}

Here, ${Q_i}$ is an $n \times {p_i}$ matrix of IVs where *n* is the sample size and ${p_i}$ is the number of IVs for *i*th gene (${g_i}$), and ${W_i}$ is a ${p_i} \times$1 vector of the estimated coefficients from the eQTL analysis. If the correlation between predicted gene expression and the actual value exceeded 0.5, we investigated the gene–OS relationship using ${\hat{g}_i}$values in a multivariable additive hazard model described earlier. The model included all predicted genes. The genes with the significant $\hat{g}$-OS relationship (*P*-value <.1) were considered as genes with a causal effect on OS.

### Replication analysis

For the replication of causal genes from the MR analysis, we performed a two-sample MR study using 602 samples in the validation cohort (296 under bevacizumab treatment and 306 under cetuximab treatment) with genotype and clinical outcome and no RNA-seq data. We first compared the clinical characteristics and demography of patients in discovery and validation cohorts [1] (Supplementary Table S1) to ensure compatibility of the cohorts. We then predicted gene expression levels of the causal genes in the validation cohort using summary statistics of eQTLs from discovery cohort as


\begin{eqnarray*}
\hat{g}_i^* = \ Q_i^*\ {\left( {Q_i^T{Q_i}} \right)^{ - 1}}Q_i^T{g_i},
\end{eqnarray*}

where ${Q_i}$ is an $n \times {p_i}$ matrix of eQTLs, where *n* is the sample size in the discovery cohort (with eQTL) and ${p_i}$ is the number of eQTLs selected as IVs for the *i*th gene (${g_i}$), and $Q_i^*$ is a ${n^*} \times {p_i}$ matrix where ${n^*}$ is the sample size in validation cohort. If the predicted expression levels ($\hat{g}_i^*$) significantly affected OS via treatment-specific additive hazard model, we considered it a replication for the one-sample MR. This analysis has some similarities with transcriptome-wide association studies (TWAS). However, the main difference is that, in the two-sample MR analysis, genetic variants as predictors satisfy MR assumptions, which might not be the case in TWAS.

### Prognostic contribution

To assess the contribution of the identified treatment-specific biomarkers to the OS model, we performed a likelihood ratio test (LRT) comparing models with and without the biomarkers while adjusting for the covariates.

## Results

In our investigation of treatment-specific biomarkers predictive of CRC treatment response, we focused on cetuximab and bevacizumab monoclonal antibodies used in the randomized phase III trial CALGB/SWOG 80405. The trial was designed to compare cetuximab, bevacizumab, or cetuximab + bevacizumab, each plus chemotherapy as first-line therapy in KRAS wild-type advanced or metastatic CRC. The discovery cohort encompassed 273 patients with pre-treatment tumor primary tissue RNA-seq and germline genotype data (Supplementary Figs S1 and S2). The validation cohort included 602 patients with germline genotype data (Supplementary Figs S1 and S2). In both cohorts, we had records of OS and conducted an MR technique, one- and two-sample MR, to identify genes with causal effects on treatment-specific OS. In one-sample MR analysis, we assessed the causal effect of the genes associated with OS. We replicated the identified associations using TCGA data. To reproduce the causal impact of genes on OS, we predicted gene expression levels by germline genotype data in the validation cohort and eQTL summary statistics from the discovery cohort [16] and performed a two-sample MR analysis. In addition, we investigated the differential expression of the identified biomarkers between colorectal tumors and normal tissue using the GSE146889 dataset from the Gene Expression Omnibus database. We finally linked the findings to CMS to investigate the genetic basis underpinning CMS [2].

**Figure 1.**
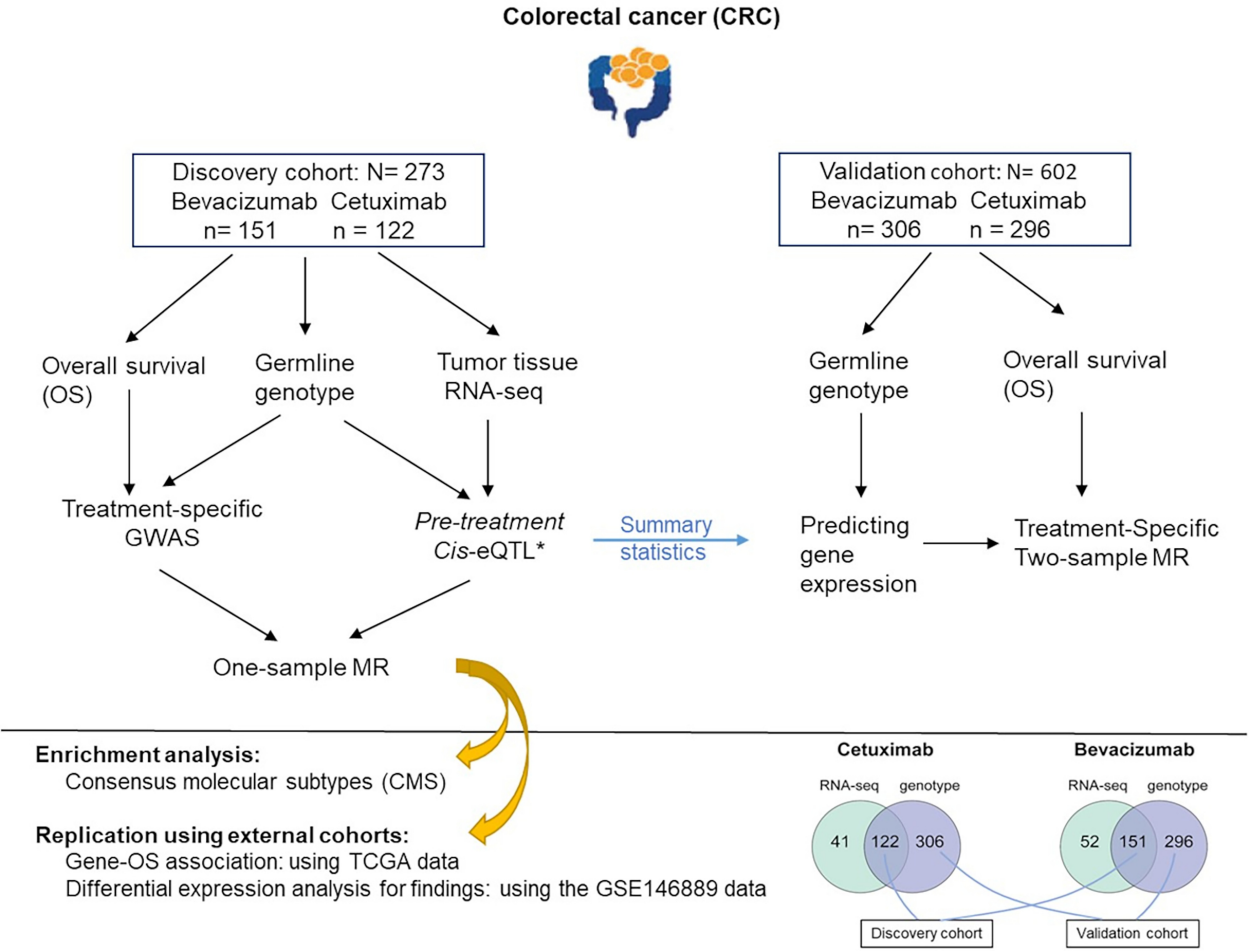
Study design and analytic workflow. The reported number of samples is after excluding patients with missing clinical outcomes or covariates. *: Published elsewhere [16] and involved *n* = 350 samples.

The analyses reviewed above were adjusted for all RAS and BRAF V600E mutation statuses along with age and gender (Fig. 1). Additionally, to enhance the precision of our findings, we accounted for the tumor microenvironment’s influence by adjusting for enriched immune cell abundances in the RNA-seq data.

### The effect of genes on treatment-specific OS

**Figure 2.**
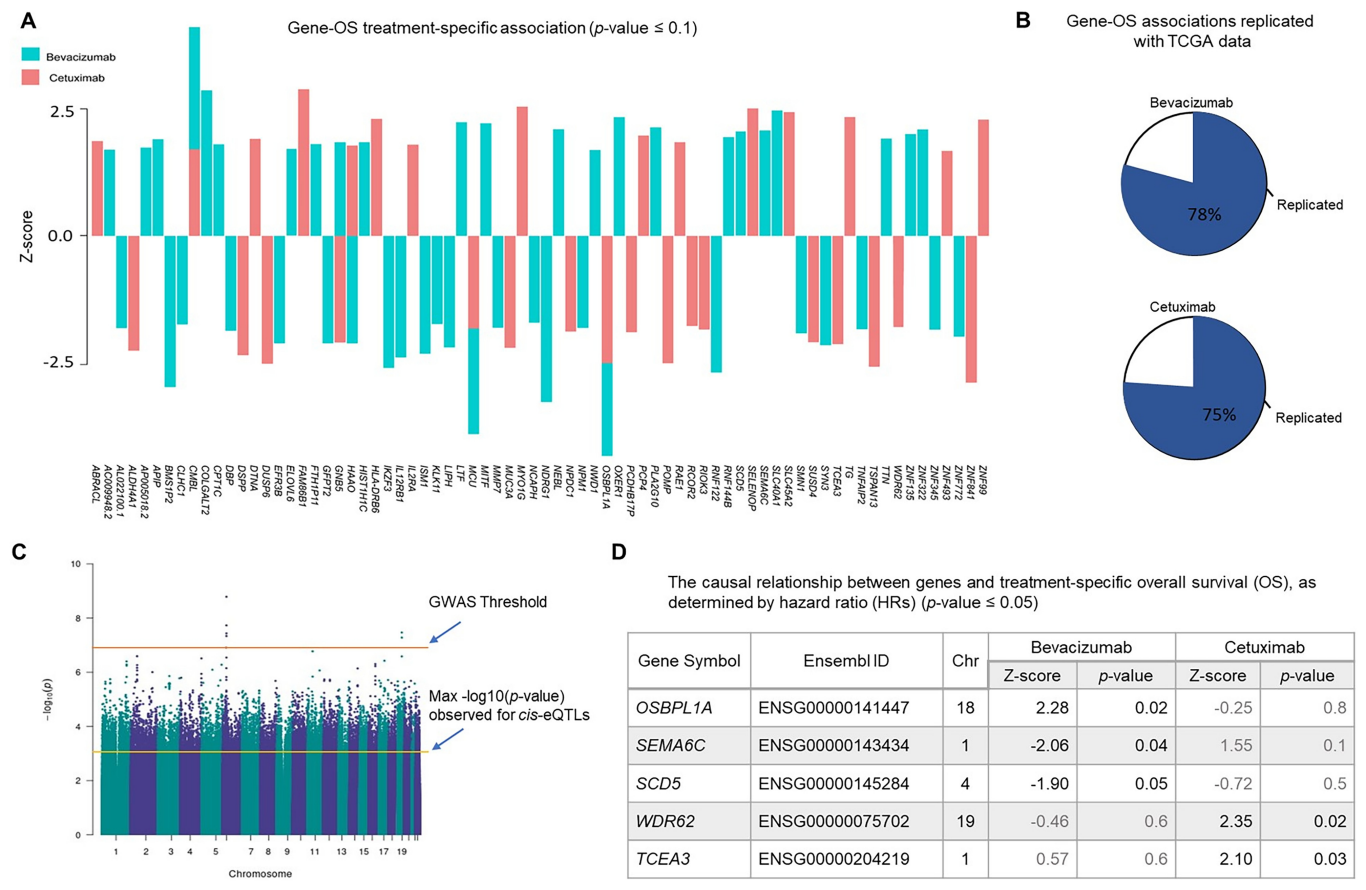
Gene–OS relationships. (**A**) The bar plot displays the *z*-scores for 79 genes significantly associated with OS (*P*-value <.1), as determined using a treatment-specific additive multivariable hazard model (see the “Materials and methods” section). (**B**) The pie charts represent the percentage of replicated gene–OS association using univariate analysis of the TCGA data. The rate of replication is relatively high even though the censoring rate of TCGA-COAD data is higher than the data in this study. (**C**) The Manhattan plot of the GWAS representing −log_10_(*P*-value) observed for genetic variants–OS associations. The GWAS threshold and the maximum −log(*P*-value) observed for the eQTL–OS associations are depicted on the plot. (**D**) Genes causally associated with treatment-specific OS based on a one-sample MR technique (*P*-value ≤.05). The *P*-values under both treatments are presented for comparison.

To identify genes with a causal effect on OS, we focused on 352 genes with eQTLs [16], since they are good candidates for MR analysis due to their higher likelihood of providing valid IVs. In the first step, we filtered these genes by investigating their association with OS using a multivariable time-variant additive hazard regression model (see the “Materials and methods” section). Out of the 352 genes with eQTLs, 79 genes were associated with OS (*P*-value <.1), including 47 under bevacizumab treatment and 32 under cetuximab (Fig. 2A and Supplementary Tables S2 and S3). We replicated the gene–OS associations using the TCGA data. The rate of replication was 78% and 75% for bevacizumab and cetuximab treatments, respectively (Fig. 2B). Note that the replication rate is relatively high although the TCGA data are not a perfect replication set for the data in this study due to the higher censoring rate of the samples (Supplementary Fig. S8).

The association between genes and OS was conducted using a multivariable time-variant additive hazard regression model, which relaxes the assumption of the time-invariant effect of the genes on OS and allows for estimating the causal effects [17] in the second step. Therefore, focusing on 79 genes associated with OS in the second step, we investigated the causal associations using a one-sample MR technique with treatment-specific OS as outcomes, gene expressions as explanatory variables (exposures), and eQTLs as IVs. To evaluate the validity of eQTLs as IVs, we assessed the lack of pleiotropic action by performing a GWAS to find the association of the eQTLs with OS. The eQTLs did not show any significant association with OS at a level of 1 × 10^−4^, which is much larger than the GWAS threshold (5 × 10^−8^) (Fig. 2C and Supplementary Fig. S9). Using the eQTLs as valid IVs, we predicted the expression levels of genes and estimated their causal effects on OS (see the “Materials and methods” section). We identified five genes with causal effect on OS (*P*-value <.05), including *OSBPL1A* (Oxysterol-Binding Protein-Like 1A), *SEMA6C*, and *SCD5* (Stearoyl-CoA Desaturase 5) under bevacizumab treatment and *WDR62* (WD repeat domain 62) and *TCEA3* (Transcription Elongation Factor A3) under cetuximab (Fig. 2D).

### Replication of the causal genes

**Figure 3.**
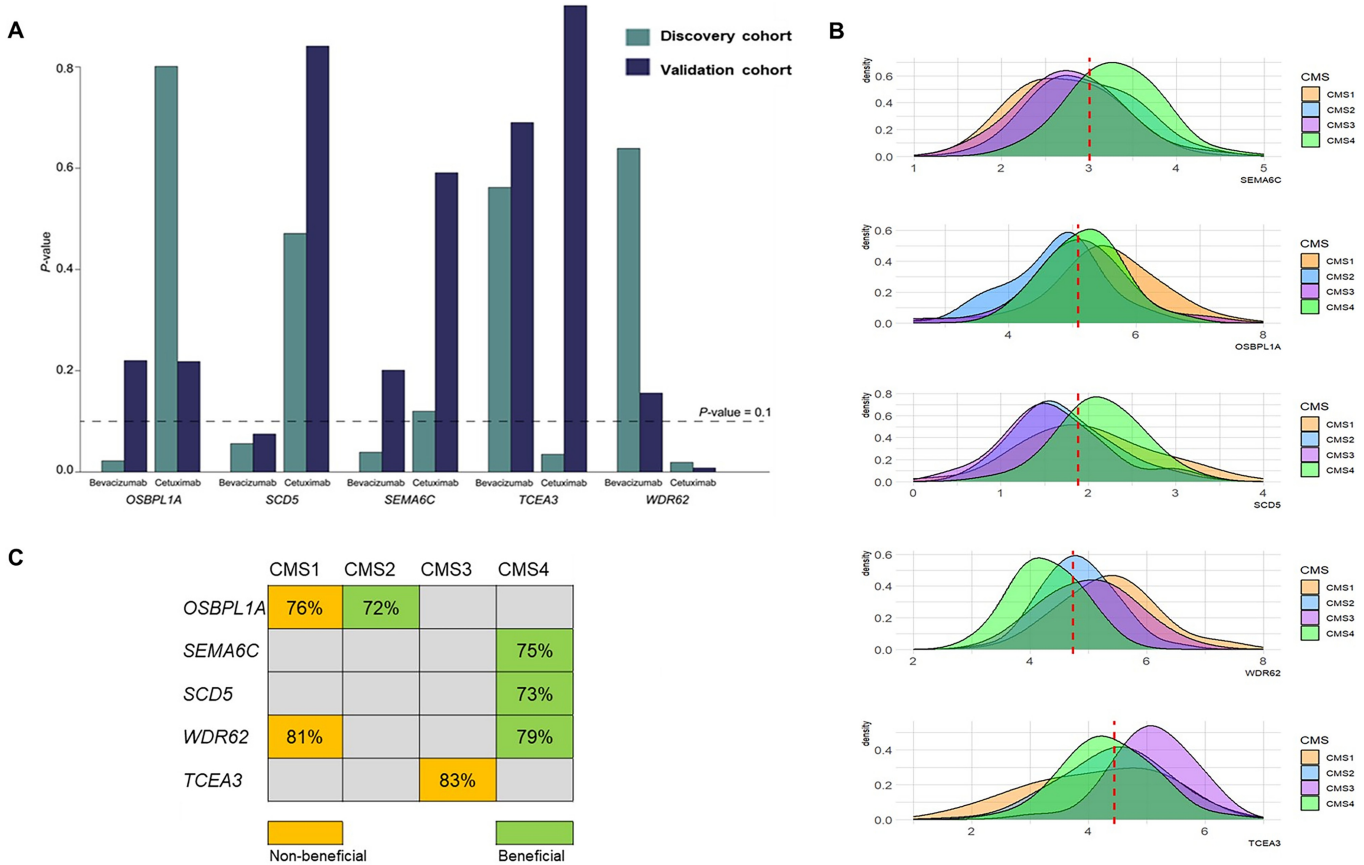
Replication of causal genes. (**A**) Replication of causal effect of genes on treatment-specific OS in a validation cohort. The cyan bars represent *P*-values calculated in the discovery cohort using one-sample MR analysis for each treatment. The navy bars represent *P*-values calculated in the validation cohort using a two-sample MR study. The dashed line shows the *P*-value threshold level for significance. (**B**) Illustrates of the subtype-specific distribution of gene expression and their enrichment. The red dashed line represents the median expression level observed across all samples. (**C**) The enrichment of gene expressions causally associated with OS in each subtype is determined based on the high percentage of patients with beneficial or non-beneficial gene expression within that specific subtype (>70%).

We reproduced the findings of one-sample MR analysis in the discovery cohort by performing a two-sample MR in the validation cohort. Using the eQTL summary statistics in CALGB-80405 [16] and the genotyping data in the validation cohort, we predicted expression levels in the validation cohort. We then estimated the effect of predicted genes on treatment-specific OS that represents the causal effect of the corresponding genes on OS (see the “Materials and methods” section). The *P*-values of significant genes–OS causal associations followed the same trend in the discovery and validation cohorts for bevacizumab versus cetuximab, except *OSBPL1A* (Fig. 3A and B). In addition, among the significant genes in the discovery cohort, all showed a slight increase in *P*-value in the validation cohort, except for *TCEA3*. Additional details are provided in Supplementary Table S4.

Furthermore, this analysis replicated the significant causal effects of *WDR62* and *SCD5*, which were identified in the discovery cohort. The negative causal effect of *WDR62* on OS under cetuximab treatment (*P*-value: .008) compared to bevacizumab (*P*-value: .16) was replicated. *WDR62* is a protein-coding gene that is implicated in the carcinogenesis of various cancers, including CRC. *WDR62* promotes tumor growth and resistance to oxaliplatin, and enhances DNA repair in CRC cells [18].

The positive causal effect of *SCD5* was also replicated, indicating its impact on OS under bevacizumab (*P*-value: .075) compared to cetuximab (*P*-value: .841). SCD5 is the stearoyl-CoA desaturating enzyme that converts saturated fatty acids to monounsaturated fatty acids. Due to its effects on fatty acid metabolism, it could potentially reduce tumor aggressiveness and promote immune activation, as reported for breast cancer [19].

In addition, we investigated whether genes causally associated with OS show differential expression between normal and tumor tissues. To assess this, we utilized GSE146889 data from the Gene Expression Omnibus database [20] as an external cohort, focusing on 24 paired samples from normal and tumor tissue of CRC patients (Supplementary Table S5). Differential expression analysis revealed significant dysregulation of *OSBPL1A* and *WDR62* (FC: .61, *P*-value: .001; FC: 4.56, *P*-value: <.0001). The expression distribution of the genes with causal impact on OS in the external replicated cohort GSE146889 is shown in Supplementary Fig. S10. Note that this analysis was not adjusted for covariates, as we did not have access to them, unlike the main analysis.

### Prognostic contribution of the replicated treatment-specific biomarkers

We further assessed the contribution of the replicated genes (*WDR62*, *SCD5*) to the prognostic models for treatment-specific OS. An LRT was performed to compare models with and without *SCD5* for patients treated with bevacizumab, and with and without *WDR62* for those treated with cetuximab. All models were adjusted for covariates, including gender, age, BRAF V600E mutations and all RAS mutation status, batch effects, and enriched cell type abundance. In both treatment groups, incorporating the respective biomarkers significantly improved the prognostic models (bevacizumab: $\chi^2_{(1)}$ = 5.99, *P-value* = .014; cetuximab: $\chi^2_{(1)}$= 3.4, *P-value* = .065), with a more pronounced enhancement observed in the bevacizumab arm.

To support the visualization of survival outcomes, we dichotomized the expression level of replicated genes into “high” and “low” groups using the median expressions as the cutoff. Kaplan–Meier (KM) estimators were then used to estimate the survival functions (Supplementary Fig. S11). It is important to note that dichotomizing continuous variables for KM analysis can obscure more nuanced associations and may not fully capture the continuous relationship between gene expression signatures and OS.

### Enrichment analysis for CRC subtypes

We investigated whether the causal genes are enriched in CMSs of CRC: CMS1 (MSI immune): tumors with high microsatellite instability (MSI-H) and strong immune activation; CMS2 (canonical): tumors with features of traditional CRC, characterized by WNT and MYC signaling activation; CMS3 (metabolic): tumors with metabolic dysregulation and KRAS mutations; CMS4 (mesenchymal): tumors with prominent stromal infiltration, inflammation, and angiogenesis [2]. We dichotomized the expression levels of the causal genes into two categories, “beneficial” and “non-beneficial,” based on their association with OS. This categorization was determined by the median expression level across all samples in our study. For genes where higher expression elongated OS, patients with expression levels above the median were classified as “beneficial,” and those below the median as “non-beneficial.” For genes where higher expression shortened OS, the classification was reversed, with patients having expression levels below the median classified as “beneficial” and those above the median as “non-beneficial.” We then calculated the proportion of patients with “beneficial” and “non-beneficial” expression levels within each subtype. We considered a threshold of 70%, and if over 70% of patients within a specific subtype showed either “beneficial” or “non-beneficial” gene expression, the subtype was considered enriched for the expression of the gene. Non-beneficial expressions of *OSBPL1A* and *WDR62* were enriched in CMS1 (rate 76% and 81%, respectively), whereas their beneficial expression levels were enriched in CMS2 (rate 72%). The CMS3 subtype exhibits an enrichment of non-beneficial levels of *TCEA3* (rate: 83%), and the CMS4 subtype exhibits an enrichment of beneficial expression levels of *SEMA6C*, *SCD5*, and *WDR62* (rate: 75%, 73%, and 79%, respectively) (Fig. 3C). The distribution of gene expressions specific to each subtype indicated an enrichment of gene expressions causally associated with OS in a specific subtype (Fig. 3B).

We evaluated the contribution of the identified novel biomarkers to the prognostic models for treatment-specific OS in the presence of CMS classification using an LRT. In the bevacizumab arm, we observed a significant improvement in model fit ($\chi _{( 3 )}^2$ = 9.68, *P*-value = .021) upon inclusion of the identified novel biomarkers (*OSBPL1A*, *SEMA6C*, and *SCD5*). For the cetuximab arm, we observed a modest improvement in model fit ($\chi _{( 2 )}^2$ = 4.2, *P-value* = .122) upon inclusion of the identified novel biomarkers (*TCEA3* and *WDR62*), though it did not reach statistical significance.

## Discussion

Here, we integrated germline genotype and RNA-seq data from a randomized phase III trial and assessed the causal effect of gene expressions on OS of patients treated with either cetuximab or bevacizumab monoclonal antibodies. We identified 47 genes associated with OS of patients treated with bevacizumab and 32 genes in cetuximab, where three genes, *OSBPL1A*, *SCD5*, and *SEMA6C*, showed causal effect on OS in the bevacizumab arm and two genes, *TCEA3* and *WDR62*, in cetuximab, respectively. Replicating these results using an external cohort represented differential expression of *OSBPL1A*, *SEMA6C*, *TCEA3*, and *WDR62*. We also replicated the causal effect of *WDR62* and *SCD5* on OS using a validation cohort with 602 additional samples.

*TCEA3* is a protein-coding gene that is involved in the regulation of transcription, particularly in the elongation phase of transcription. Our study showed that the overexpression of *TCEA3* reduces the OS of patients treated with cetuximab. Additionally, >80% of patients with the CMS3 subtype exhibited high expression of *TCEA3*. In consistence with this finding here, the inhibition of *TCEA3* is suggested as an effective agent to enhance various chemotherapeutics-induced pyroptosis [21].

*OSBPL1A* is a gene that encodes a protein involved in the transport and regulation of lipids, specifically oxysterols. Oxysterols are oxidized derivatives of cholesterol and play roles in various cellular processes, including cholesterol homeostasis and lipid signaling, which can affect the development and progression of various cancers, such as CRC. We observed a negative impact of high expression of this gene on the OS of patients treated with bevacizumab, which was enriched among patients with the CMS1 subtype.

Our study revealed that the upregulation of *SCD5* potentially elongates OS in patients treated with bevacizumab, which is a VEGF inhibitor. *SCD5* is required for lipid synthesis, a key regulator of energy metabolism. Dysregulation of *SCD5* may play a role in dyslipidemia, characterized by abnormal lipid levels in the blood. Furthermore, dyslipidemia has been associated with CRC and tumor progression [22]. Moreover, the risk of dyslipidemia with the use of VEGF/VEGFR inhibitors has been observed [23]. Therefore, understanding the interplay between *SCD5*, dyslipidemia, and VEGF inhibition could yield valuable insights into CRC pathogenesis and therapeutic strategies.

Furthermore, our study showed that the overexpression of *WDR62* shortened the OS of patients treated with cetuximab and may contribute to cetuximab-resistant CRC. WDR62 is a scaffold protein involved in several important cellular processes, such as forming of the structure of the cell nucleus, regulation of gene expression, and ensuring proper division of cells during cell division. *WDR62* coordinates TNFα receptor signaling pathway to the JNK activation [24]. In addition, there is a complex interaction between TNFα and EGFR, which is the targeted pathway of cetuximab [25]. WDR62 also contributes to multidrug resistance of gastric cancer through activation of MAPK signaling [18]. In our study, overexpression of *WDR62* is enriched in CMS1, whereas its underexpression is enriched in CMS4. Therefore, *WDR62* may be instrumental in understanding interactions between JNK signaling and specific CRC subtypes, potentially contributing to the development of more personalized and effective treatment strategies.

Collectively, our study underscores the utility of incorporating genotype data into gene–OS investigations to discover novel biomarkers with causal associations with OS in observational studies and to shed light on mechanisms underlying response to specific treatments.

## Supplementary Material

lqaf053_Supplemental_Files

## Data Availability

The gene expression data used in this study are publicly available in Gene Expression Omnibus at GSE196576. The genotype data can be accessed upon approval from the National Institutes of Health (NIH). For the external cohorts, the TCGA data were obtained using the TCGAbiolinks R package and The Human Protein Atlas, and the second external cohort was downloaded from GEO (GSE146889). The code is accessible via GitHub repository at https://github.com/AkramYazdani/MR/blob/main/R_Script.txt, and Harvard Dataverse, https://doi.org/10.7910/DVN/IQHEGF.
