# Supplementary material for: Novel treatment-specific causal biomarkers for colorectal cancer by omics integration
Source: NAR Genom Bioinform. 2025 Jun 19;7(2):lqaf053. doi: 10.1093/nargab/lqaf053 (PMC12204401; doi:10.1093/nargab/lqaf053)
Supplement: lqaf053_Supplemental_Files [file lqaf053_supplemental_files.zip › Supplementary_AY_etal.docx]

**Supplementary**

**Novel treatment-specific causal biomarkers for colorectal cancer by omics integration**

Akram Yazdani^*^, Azam Yazdani, Raul Mendez-Giraldez, Gianluigi Pillonetto, Esmat Samiei, Reza Hadi, Heinz-Josef Lenz, Alan P Venook, Ahmad Samiei, Andrew B Nixon, Joseph A Lucci 3rd, Scott Kopetz, Monica M Bertagnolli, Charles M Perou, Federico Innocenti

**Supplementary figures**

**Figure S1.** Consort chart for germline genotype data

**
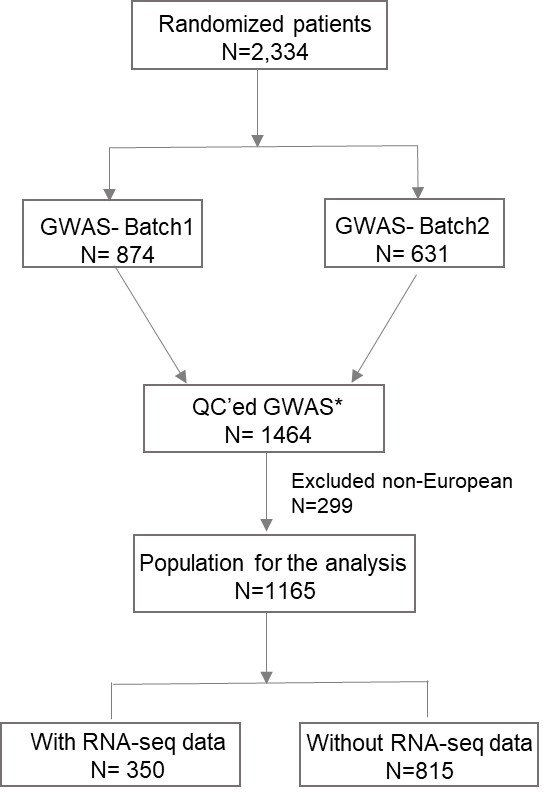
**

**
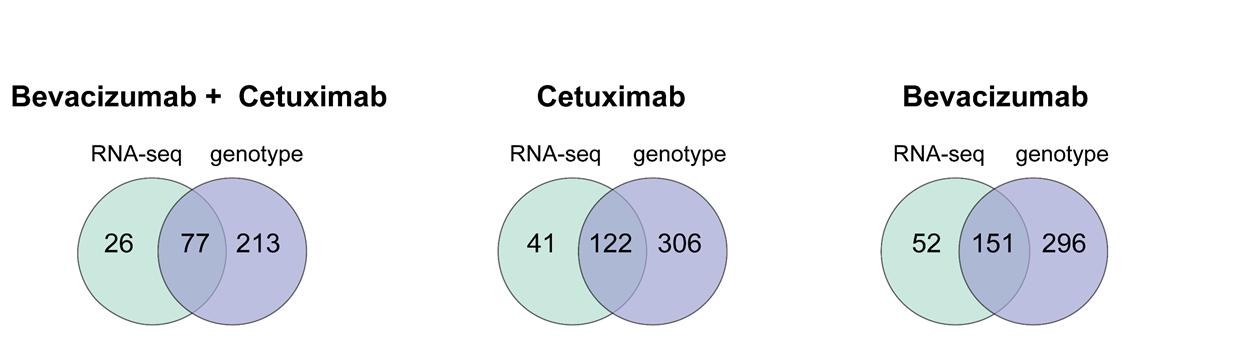
**

**Figure S2.** Consort chart for RNA-seq data

**
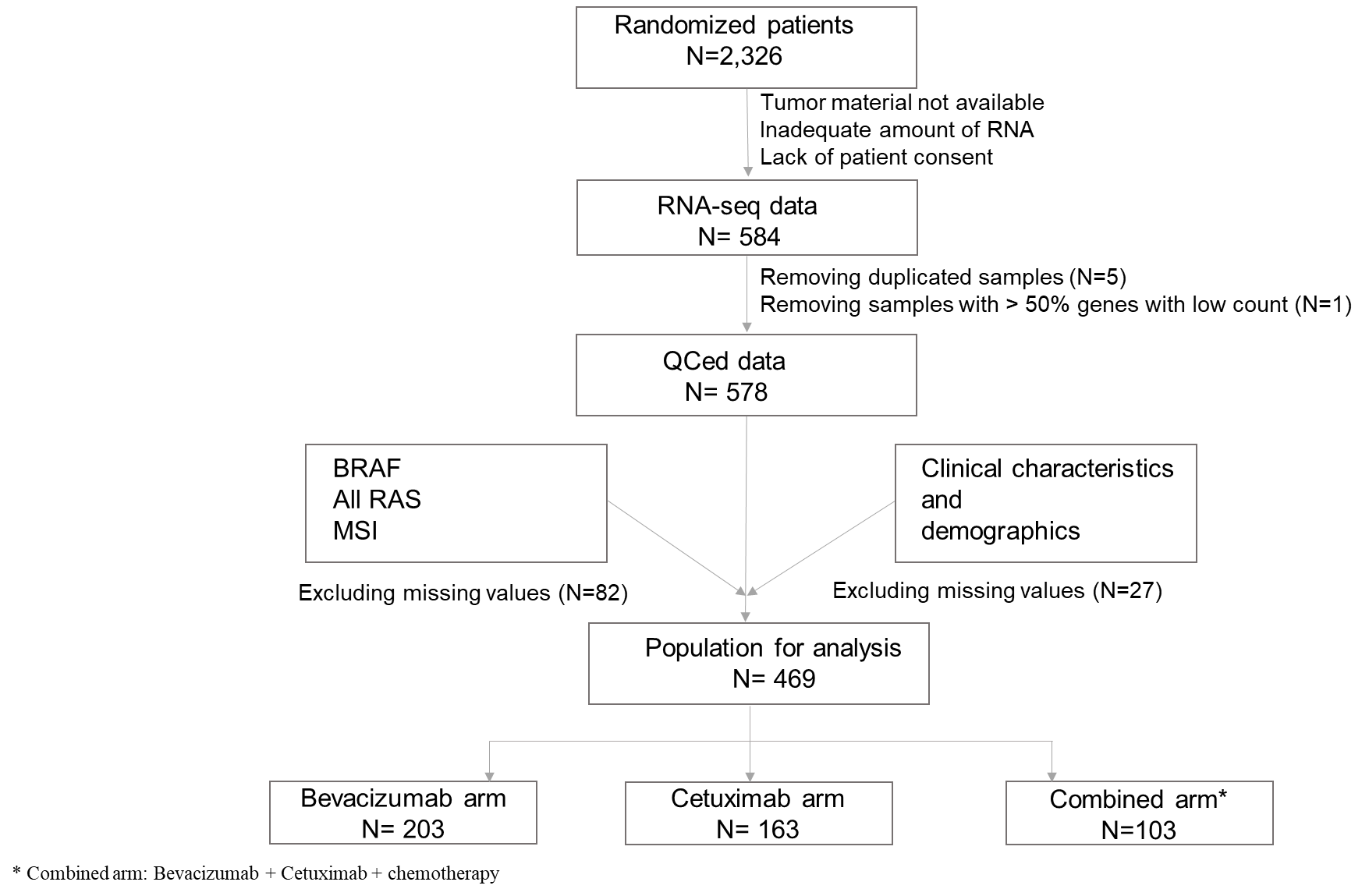
**

*Combined arm: Bevacizumab + Cetuximab + chemotherapy

**Figure S3.** Principal component analysis to assess presents of batch effect on RNA-seq


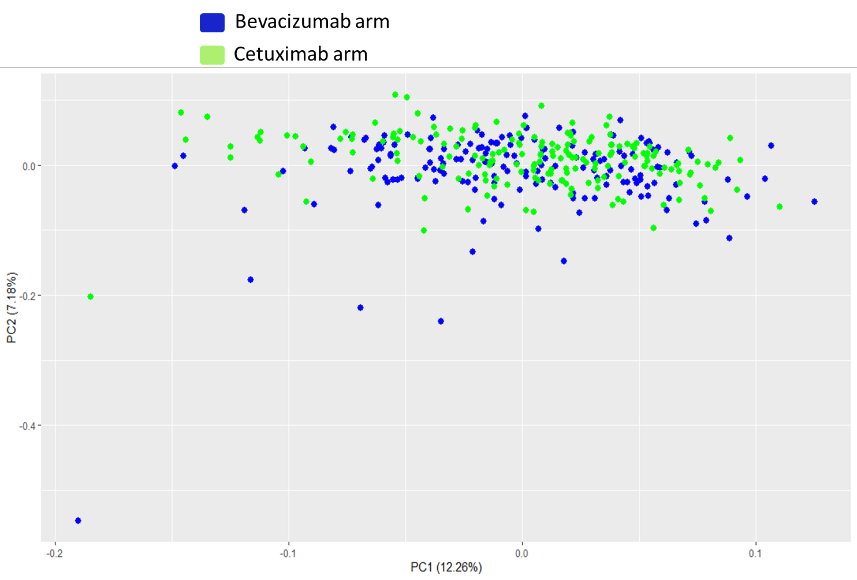


**
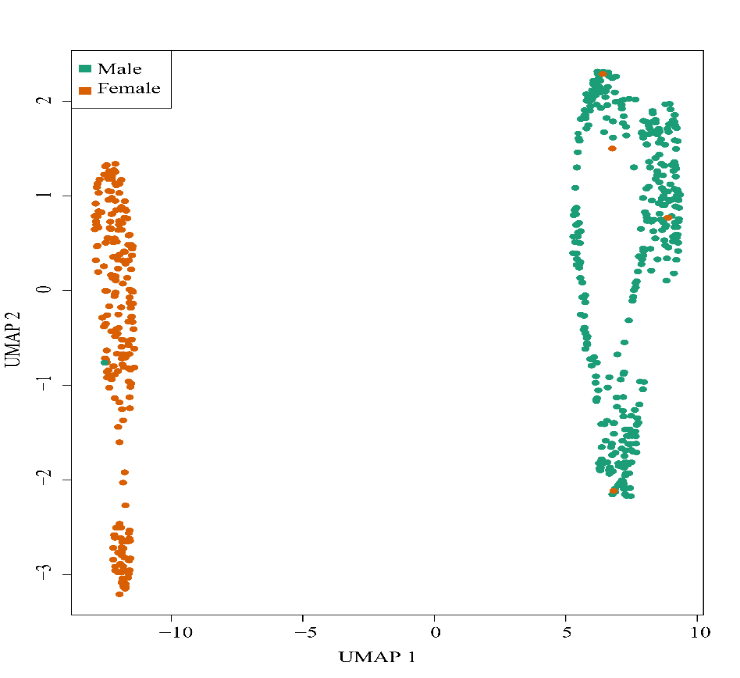
Figure S4**. Gender verification using k means clustering of samples based on expression of genes in chromosome Y.

**Figure S5.** Histogram of the distance of each *cis*-eQTL from TSS of the gene

**
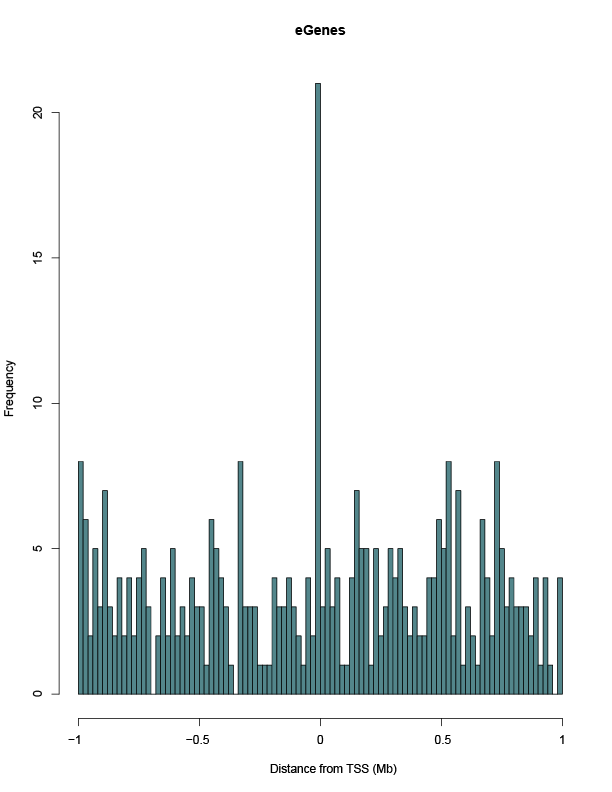
**

**Figure S6.** *P*-values of *cis*-eQTL vs the distance from TSS of the gene

**
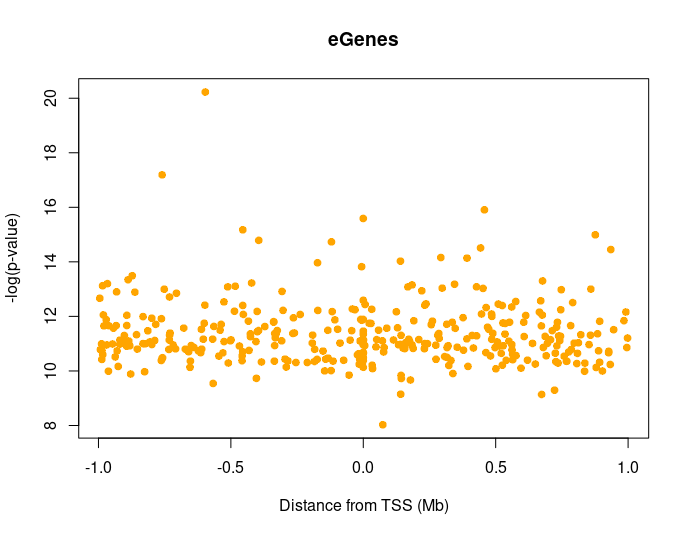
**

**Figure S7.** The correlation of first principal components of genotyped data with other covariates

**
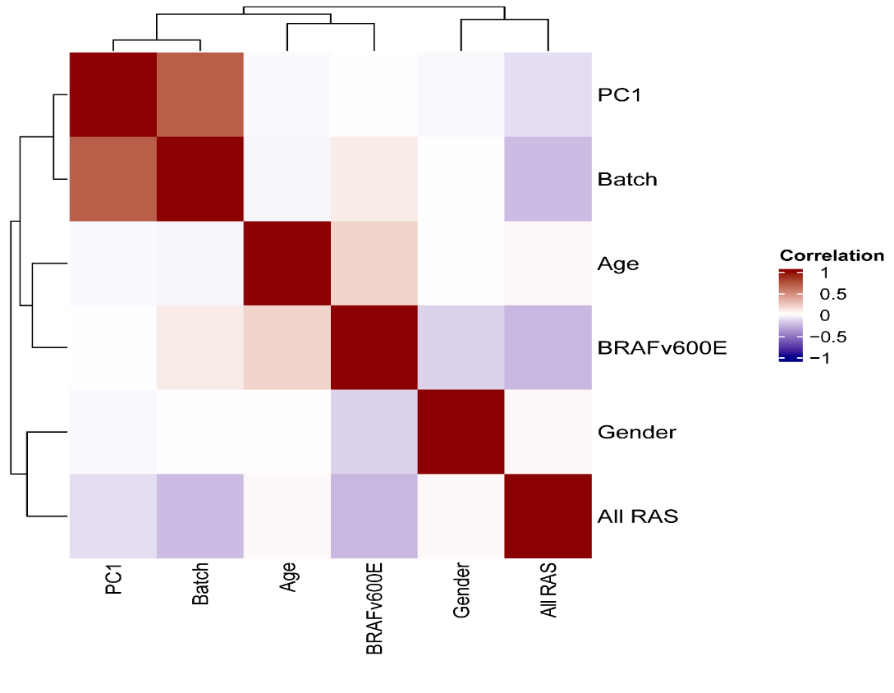
**


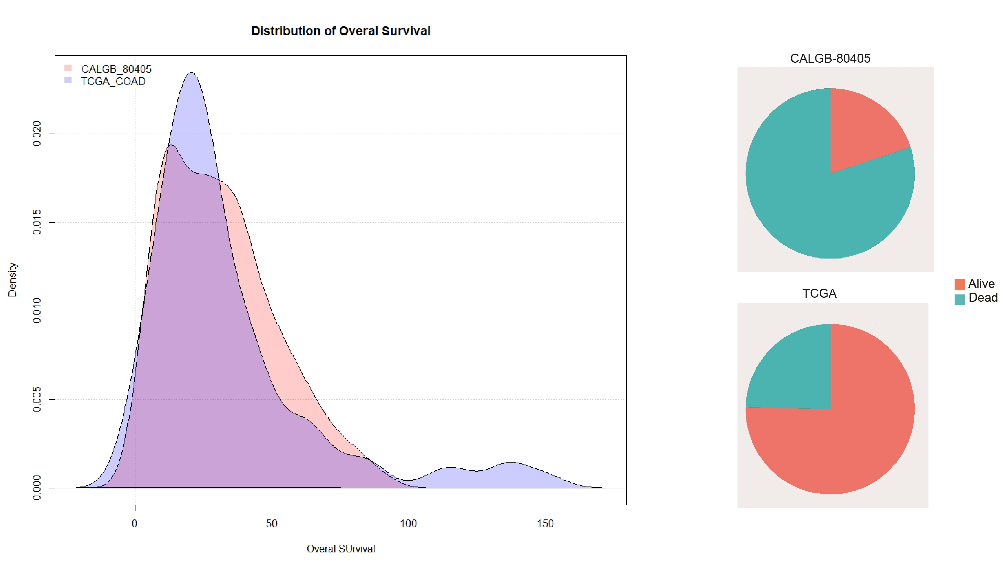
**Figure S8.** Comparison of the data from CALGB-80405 and TCGA in terms of distribution of OS and events.

**Figure S9.** Assessing the pleiotropy of *cis*-eQTLs to find valid IVs for MR analysis. Left panel: The Manhattan plot shows that *cis*-eQTLs do not significantly affect OS. Right panel: The histogram shows how the *p*-values corresponding to IVs are distributed.

**
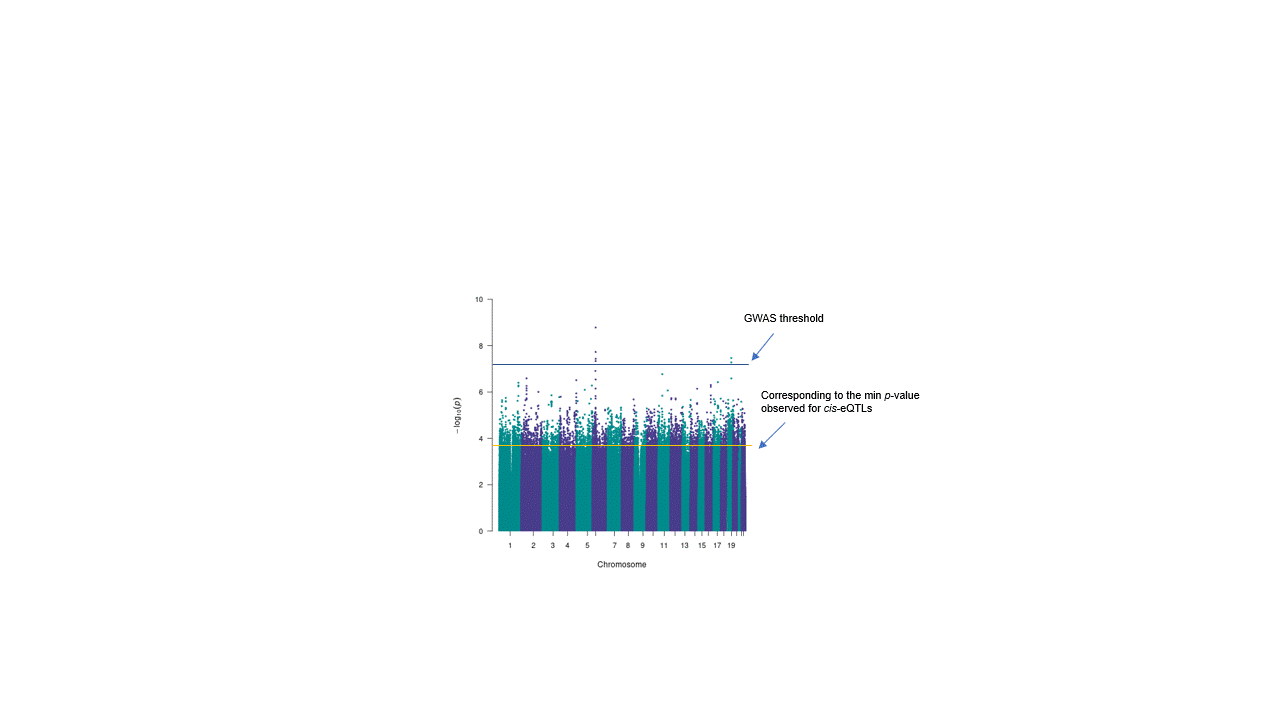

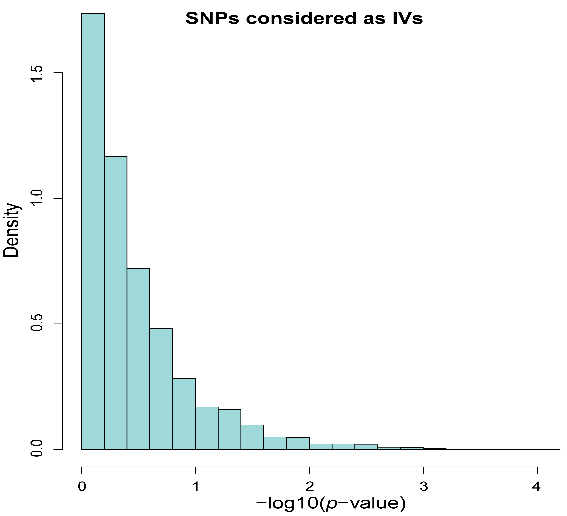
**

**Figure S10.**  The distribution of quantile-normalized gene expression for genes with a causal impact on OS in the external replicated cohort GSE146889.

**Figure S11.** Kaplan-Meier plots for replicated genes with causal effect on treatment specific OS. The blue curve represents OS for patients with a high expression, while the orange curve represents those with a low expression level, with the cutoff defined based on the median expression value.

**Supplemantery Tables**

**Table S1.** Patient clinical demographics and characteristics in CALGB-80405 trial. Comparisons are between the population with the RNA-seq data and the population without it.

|  | | | | |  |
| --- | --- | --- | --- | --- | --- |
|  | | **Population without**  **RNAseq   (N=1748)** | **Population with RNAseq   (N=578)** | **Total (N=2326)** | **P value** |
| **Age (years)** | |  |  |  | 0.01^1^ |
| Median | | 58.91 | 60.36 | 59.14 |  |
| Range | | (20.77-89.51) | (23.99-83.45) | (20.77-89.51) |  |
|  | |  |  |  |  |
| **Arm** | |  |  |  | 0.49^2^ |
| Bevacizumab+chemotherapy | | 662 (37.9%) | 235 (40.6%) | 897 (38.6%) |  |
| Cetuximab+chemotherapy | | 681 (39.0%) | 216 (37.4%) | 897 (38.6%) |  |
| Bevacizumab/cetuximab+chemotherapy | | 405 (23.1%) | 127 (22.0%) | 532 (22.8%) |  |
|  | |  |  |  |  |
| **Chemotherapy** | |  |  |  | 0.275^2^ |
| FOLFOX | | 1359 (77.7%) | 436 (75.4%) | 1795 (76.2%) |  |
| FOLFIRI | | 389 (22.3%) | 142 (24.5%) | 531 (22.1%) |  |
|  | |  |  |  |  |
| **Prior Adjuvant Chemotherapy** | |  |  |  | 0.123^2^ |
| No | | 1486 (85.0%) | 507 (87.7%) | 1993 (85.7%) |  |
| Yes | | 262 (15.0%) | 71 (12.3%) | 333 (14.3%) |  |
|  | |  |  |  |  |
| **Gender** | |  |  |  | 0.08^2^ |
| Male | | 1000 (57.2%) | 355 (61.4%) | 1355 (58.3%) |  |
| Female | | 748 (42.8%) | 223 (38.6%) | 971 (41.7%) |  |
|  | |  |  |  |  |
| **Race** | |  |  |  | <0.0001^2^ |
| Unknown | | 51 (2.9%) | 3 (0.5%) | 54 (2.3%) |  |
| White | | 1396 (79.9%) | 500 (86.5%) | 1896 (81.7%) |  |
| African American | | 225 (12.9%) | 53 (9.2%) | 278 (12.0%) |  |
| Asian | | 60 (3.4%) | 13 (2.2%) | 73 (3.1%) |  |
| Native Hawaiian or Pacific Islander | | 7 (0.4%) | 0 (0.0%) | 7 (0.3%) |  |
| American Indian or Alaska Native | | 8 (0.5%) | 3 (0.005%) | 11 (0.5%) |  |
| More than one race | | 1 (0.1%) | 5 (0.8%) | 6 (0.3%) |  |
|  | |  |  |  |  |
| **ECOG Performance Status** | |  |  |  | 0.461^2^ |
| 0 | | 1030 (58.9%) | 331 (57.2%) | 1361 (58.5%) |  |
| 1 | | 715 (40.9%) | 247 (42.7%) | 962 (41.4%) |  |
| 2 | | 3 (0.2%) | 0 (0.0%) | 3 (0.1%) |  |
|  | |  |  |  |  |
| **Overall Survival (months)** | |  |  |  | 0.04^3^ |
| Median (95% CI) | | 24.5 (23.6-25.8) | 27.5 (25.6-30.2) | 25.4 (24.4-26.4) |  |
|  | |  |  |  |  |
| **Progression-Free Survival (months)** | |  |  |  | 0.005^3^ |
| Median (95% CI) | | 9.66 (9.35-10.1) | 10.6 (9.59-11.3) | 9.82 (9.49-10.2) |  |
| ^1^Kruskal Wallis    ^2^Chi-Square    ^3^Log-rank | |  |  |  |  |

**Table S2.** Summary result of additive hazard model for samples under bevacizumab treatment.

| Gene Symbol | Ensembl ID | Chr | Z-score | *p*-value | TCGA  *p*-value |
| --- | --- | --- | --- | --- | --- |
| *AC009948.2* | ENSG00000270277 | 2 | -1.677 | 0.0936 | --- |
| *AL022100.1* | ENSG00000227722 | 1 | 1.794 | 0.073 | --- |
| *AP005018.2* | ENSG00000254851 | 11 | -1.715 | 0.086 | --- |
| *APIP* | ENSG00000149089 | 11 | -1.879 | 0.060 | 0.05 |
| *BMS1P2* | ENSG00000251079 | 10 | 2.935 | 0.003 | --- |
| *CLHC1* | ENSG00000162994 | 2 | 1.722 | 0.085 | 0.034 |
| *CMBL* | ENSG00000164237 | 5 | -2.372 | 0.018 | 0.0081 |
| *COLGALT2* | ENSG00000198756 | 1 | -2.825 | 0.005 | 0.00006 |
| *CPT1C* | ENSG00000169169 | 19 | -1.780 | 0.0750 | 0.010 |
| *DBP* | ENSG00000105516 | 19 | 1.842 | 0.065 | 0.0022 |
| *EFR3B* | ENSG00000084710 | 2 | 2.088 | 0.037 | 0.21 |
| *ELOVL6* | ENSG00000170522 | 4 | -1.698 | 0.089 | 0.00067 |
| *FTH1P11* | ENSG00000237264 | 8 | -1.786 | 0.074 | --- |
| *GFPT2* | ENSG00000131459 | 5 | 2.086 | 0.036 | 0.019 |
| *GNB5* | ENSG00000069966 | 15 | -1.817 | 0.069 | 0.0020 |
| *HAAO* | ENSG00000162882 | 2 | 2.089 | 0.037 | 0.016 |
| *HIST1H1C* | ENSG00000187837 | 6 | -1.821 | 0.069 | 0.013 |
| *IKZF3* | ENSG00000161405 | 17 | 2.564 | 0.010 | 0.061 |
| *IL12RB1* | ENSG00000096996 | 19 | 2.360 | 0.018 | 0.0091 |
| *ISM1* | ENSG00000101230 | 20 | 2.287 | 0.022 | 0.0044 |
| *KLK11* | ENSG00000167757 | 19 | 1.711 | 0.087 | 0.13 |
| *LIPH* | ENSG00000163898 | 3 | 2.165 | 0.030 | 0.14 |
| *LTF* | ENSG00000012223 | 3 | -2.207 | 0.027 | 0.059 |
| *MCU* | ENSG00000156026 | 10 | 2.051 | 0.040 | 0.030 |
| *MITF* | ENSG00000187098 | 3 | -2.187 | 0.029 | 0.030 |
| *MMP7* | ENSG00000137673 | 11 | 1.782 | 0.075 | 0.042 |
| *NCAPH* | ENSG00000121152 | 2 | 1.686 | 0.092 | 0.029 |
| *NDRG1* | ENSG00000104419 | 8 | 3.227 | 0.001 | 0.085 |
| *NEBL* | ENSG00000078114 | 10 | -2.069 | 0.039 | 0.034 |
| *NPM1* | ENSG00000181163 | 5 | 1.788 | 0.074 | 0.022 |
| *NWD1* | ENSG00000188039 | 19 | -1.671 | 0.095 | 0.031 |
| *OSBPL1A* | ENSG00000141447 | 18 | 1.803 | 0.071 | 0.0098 |
| *OXER1* | ENSG00000162881 | 2 | -2.306 | 0.021 | 0.031 |
| *PLA2G10* | ENSG00000069764 | 16 | -2.108 | 0.035 | 0.067 |
| *RNF122* | ENSG00000133874 | 8 | 2.652 | 0.008 | 0.021 |
| *RNF144B* | ENSG00000137393 | 6 | -1.917 | 0.055 | 0.16 |
| *SCD5* | ENSG00000145284 | 4 | -2.035 | 0.042 | 0.29 |
| *SEMA6C* | ENSG00000143434 | 1 | -2.047 | 0.041 | 0.0016 |
| *SLC40A1* | ENSG00000138449 | 2 | -2.435 | 0.015 | 0.094 |
| *SMN1* | ENSG00000172062 | 5 | 1.892 | 0.058 | 0.32 |
| *SYN3* | ENSG00000185666 | 22 | 2.120 | 0.034 | 0.0022 |
| *TNFAIP2* | ENSG00000185215 | 14 | 1.811 | 0.070 | 0.0016 |
| *TTN* | ENSG00000155657 | 2 | -1.888 | 0.059 | 0.18 |
| *ZNF135* | ENSG00000176293 | 19 | -1.977 | 0.048 | 0.065 |
| *ZNF322* | ENSG00000181315 | 6 | -2.068 | 0.039 | 0.052 |
| *ZNF345* | ENSG00000251247 | 19 | 1.820 | 0.069 | 0.22 |
| *ZNF772* | ENSG00000197128 | 19 | 1.957 | 0.050 | 0.11 |

**Table S3.** Summary result of additive hazard model for samples under cetuximab treatment.

| Gene Symbol | Ensembl ID | Chr | Z-score | *p*-value | TCGA  *p*-value |
| --- | --- | --- | --- | --- | --- |
| *ABRACL* | ENSG00000146386 | 6 | -1.843 | 0.065 | 0.093 |
| *ALDH4A1* | ENSG00000159423 | 1 | 2.228 | 0.026 | 0.014 |
| *CMBL* | ENSG00000164237 | 5 | -1.682 | 0.093 | 0.0081 |
| *DSPP* | ENSG00000152591 | 4 | 2.317 | 0.021 | --- |
| *DTNA* | ENSG00000134769 | 18 | -1.883 | 0.060 | 0.0031 |
| *DUSP6* | ENSG00000139318 | 12 | 2.483 | 0.013 | 0.058 |
| *FAM86B1* | ENSG00000186523 | 8 | -2.851 | 0.004 | 0.28 |
| *GNB5* | ENSG00000069966 | 15 | 2.070 | 0.038 | 0.0020 |
| *HAAO* | ENSG00000162882 | 2 | -1.753 | 0.080 | 0.016 |
| *HLA-DRB6* | ENSG00000229391 | 6 | -2.273 | 0.023 | --- |
| *IL2RA* | ENSG00000134460 | 10 | -1.772 | 0.076 | 0.017 |
| *MCU* | ENSG00000156026 | 10 | 1.801 | 0.072 | 0.030 |
| *MUC3A* | ENSG00000169894 | 7 | 2.175 | 0.030 | 0.17 |
| *MYO1G* | ENSG00000136286 | 7 | -2.512 | 0.012 | 0.0052 |
| *NPDC1* | ENSG00000107281 | 9 | 1.858 | 0.063 | 0.000020 |
| *OSBPL1A* | ENSG00000141447 | 18 | 2.471 | 0.013 | 0. 0098 |
| *PCDHB17P* | ENSG00000255622 | 5 | 1.872 | 0.061 | --- |
| *PCP4* | ENSG00000183036 | 21 | -1.950 | 0.051 | 0.053 |
| *POMP* | ENSG00000132963 | 13 | 2.472 | 0.013 | 0.23 |
| *RAE1* | ENSG00000101146 | 20 | -1.819 | 0.069 | 0.045 |
| *RCOR2* | ENSG00000167771 | 11 | 1.746 | 0.081 | 0.041 |
| *RIOK3* | ENSG00000101782 | 18 | 1.817 | 0.069 | 0.24 |
| *SELENOP* | ENSG00000250722 | 5 | -2.477 | 0.013 | 0.0043 |
| *SLC45A2* | ENSG00000164175 | 5 | -2.405 | 0.016 | 0.35 |
| *SUSD4* | ENSG00000143502 | 1 | 2.065 | 0.039 | 0.0013 |
| *TCEA3* | ENSG00000204219 | 1 | 2.103 | 0.035 | 0.092 |
| *TG* | ENSG00000042832 | 8 | -2.307 | 0.021 | 0.00074 |
| *TSPAN13* | ENSG00000106537 | 7 | 2.541 | 0.011 | 0.15 |
| *WDR62* | ENSG00000075702 | 19 | 1.777 | 0.076 | 0.032 |
| *ZNF493* | ENSG00000196268 | 19 | -1.650 | 0.099 | 0.13 |
| *ZNF841* | ENSG00000197608 | 19 | 2.850 | 0.004 | 0.036 |
| *ZNF99* | ENSG00000213973 | 19 | -2.257 | 0.024 | 0.042 |

**Table S4.** Replication of discovery cohort findings in the validation cohort.

| Gene Symbol | Ensembl ID | Chr | Bevacizumab | | Cetuximab | | | |
| --- | --- | --- | --- | --- | --- | --- | --- | --- |
|  |  |  | **Z-score** | ***p*-value** | | **Z-score** | | ***p*-value** |
| *OSBPL1A* | ENSG00000141447 | 18 | -1.226 | 0.22 | | | 1.23 | 0.218 |
| *SEMA6C* | ENSG00000143434 | 1 | 1.28 | 0.201 | | | 0.537 | 0.591 |
| *SCD5* | ENSG00000145284 | 4 | **-1.781** | **0.075** | | | -0.2 | 0.841 |
| *WDR62* | ENSG00000075702 | 19 | -1.42 | 0.156 | | | **2.646** | **0.008** |
| *TCEA3* | ENSG00000204219 | 1 | -0.398 | 0.69 | | | 0.099 | 0.921 |

**Table S5.** Sample labels of 24 paired normal and tumor tissue samples from colorectal cancer patients in GSE146889, used as an external replication cohort.

| Sample ID |
| --- |
| MSI_MLH1HM_11 |
| MSI_MLH1HM_15 |
| MSI_MLH1HM_12 |
| MSI_MLH1HM_13 |
| MSI_MSH2_1 |
| MSI_MSH2_2 |
| MSI_MSH2_4 |
| MSI_MSH2_5 |
| MSI_MSH2_6 |
| MSI_MSH2_7 |
| MSI_MSH2_9 |
| MSI_PMS2_1 |
| MSI_PMS2_10 |
| MSI_PMS2_13 |
| MSI_PMS2_14 |
| MSI_PMS2_15 |
| MSI_PMS2_3 |
| MSI_PMS2_4 |
| MSI_PMS2_5 |
| MSI_PMS2_6 |
| MSI_PMS2_7 |
| MSI_MSH2_10 |
| MSI_MSH6_12 |
| MSI_PMS2_16 |
